# Precise tumor size measurement under constant pressure by novel real-time micro-electro-mechanical-system hood for proper treatment (with videos)

**DOI:** 10.1007/s00464-014-3642-3

**Published:** 2014-07-04

**Authors:** Hirohito Mori, Hidekuni Takao, Hideki Kobara, Noriko Nishiyama, Shintaro Fujihara, Tae Matsunaga, Maki Ayaki, Tsutomu Masaki

**Affiliations:** 1 Department of Gastroenterology and Neurology, Kagawa University, 1750-1 Ikenobe, Miki, Kita, Kagawa 761-0793 Japan; 2 Department of Intelligent Mechanical Systems Engineering, Kagawa University, 1750-1 Ikenobe, Miki, Kita, Kagawa 761-0793 Japan

**Keywords:** Correct tumor size, Micro-electro-mechanical-system hood, Real-time monitoring, Objective evaluation

## Abstract

**Background:**

Tumor size determination is subject to the measurement method used by endoscopists and is especially dependent on the air quantity. As the intraluminal pressure must be measured objectively to obtain an accurate tumor size measurement, insufflation can affect the results. Thus, we examined the utility of a micro-electro-mechanical-system (MEMS) pressure sensor hood.

**Methods:**

Twenty consecutive air insufflation/deflation tests were performed in vivo using a dog’s stomach. Correlations between the actual pressure measured and the signal strength of the MEMS hood were measured. We marked 2 points 20 mm on the antrum and another 3 points, with insufflation corresponding to the maximum stable distance of two markings. We performed five insufflation/deflation tests to obtain the relationship between pressure and distances to accurately measure the distance under constant pressure.

**Results:**

In the air insufflation/deflation test performed 20 consecutive times, the MEMS hood signal strength (V) and the pressure measurement sensor values (mmHg) showed good correlation. There was good correlation between intraluminal pressures of 2.5–40 mmHg and the two marking distances on the antrum (correlation coefficient 0.952) (*P* < 0.05). However, once the intraluminal pressure reached a certain level (40 mmHg), expansion of the two marking distances ceased. The same measurements were conducted on the greater curvatures of the lower body and middle body and on the lesser curvature of the lower body.

**Conclusions:**

Correct tumor size measurements using a MEMS hood enable a more accurate diagnosis, which can be used to develop suitable treatment strategies.

**Electronic supplementary material:**

The online version of this article (doi:10.1007/s00464-014-3642-3) contains supplementary material, which is available to authorized users.

A micro-electro-mechanical-system (MEMS) is a device in which an actuator and an electronic circuit are integrated on a single silicon substrate of which structure is not only very simple but also sensitivity and rate of air pressure detection is very high. The actuator converts the input energy into physical kinetic motion and is a mechanical component that constitutes a machine/electric circuit. There has been progress in research and development aimed at producing medical devices, such as sensors, and in recent years, MEMS, which has an ultra-compact size, has also been used in medical devices [1, 2]. When esophagogastroduodenoscopy (EGD) is performed for medical examination and treatment, the endoscopist who performs the examination insufflates or deflates air by manually pressing the “air deflation”/“air insufflation” button on the operating unit to obtain endoscopic findings and decide on the treatments. However, occasionally, excessive air is insufflated into the patient’s digestive tract, which may cause abdomen distension, abdominal distress, and even Mallory Weiss syndrome [3]. Furthermore, in early esophageal, gastric, and colon cancers, it is very important for endoscopists to accurately measure tumor size to determine the most suitable treatment strategy. In Japan, the indications for endoscopic submucosal dissection (ESD) are defined by the Gastric Cancer Treatment guidelines of the Japanese Gastric Cancer Association (JGCA) and have been expanding. The expanding indications of ESD are strictly defined and depend on tumor size as follows: intra-mucosal differentiated adenocarcinoma of any size without an ulcer, SM1-invaded differentiated adenocarcinoma of less than 30 mm in diameter, and intra-mucosal undifferentiated or poorly differentiated adenocarcinoma of less than 20 mm in diameter.

Current digestive endoscope models are not equipped with systems that enable the real-time measurement of the air pressure inside the digestive tract. Therefore, the insufflated air volume and measurement of tumor size are based on the endoscopist’s subjective judgment and experience. As a result, excessive insufflation may occur accidentally, which may cause the measurement of tumor size to be inaccurate [4]. In Japan, although the indication of ESD is strongly addressed and defined in accordance with the maximum diameter of the tumor, we have no objective method for accurately assessing tumor size. Therefore, we started to develop an innovative new hood with a pressure-sensitive MEMS sensor attached to the tip of a flexible endoscope that can conduct real-time monitoring of intra-luminal pressure. This hood would not only prevent excessive air insufflation but would also enable the endoscopist to accurately measure the tumor size under constant pressure by real-time monitoring. We then examined whether a hood equipped with a MEMS pressure sensor could result in safer examinations and more accurate tumor size measurements, making it suitable as a treatment strategy in early esophageal, gastric, and colon cancers.

## Materials and methods

For the prototype MEMS hood, we developed an extremely minimal MEMS base and embedded it into the wall of a hood (patent number: JPN 2012-224305). After performing several basic ex vivo experiments, we conducted in vivo experiments in a dog to monitor the real-time intra-luminal pressure and to accurately measure the tumor diameter.

A 12-month-old female Beagle dog was used in this study (Hokuzan Rabesu Co., Nagano, Japan). Animal experiments were performed in the Preclinical Animal Laboratory of Kagawa University, Japan. A dog was maintained in a pathogen-free facility under controlled temperature (24 ± 2 °C) and humidity (55 ± 5 %), with a 12-h light/dark cycle. All experimental procedures were performed according to the guidelines for the care and use of animals as established by Kagawa University.

**Fig. 1:**
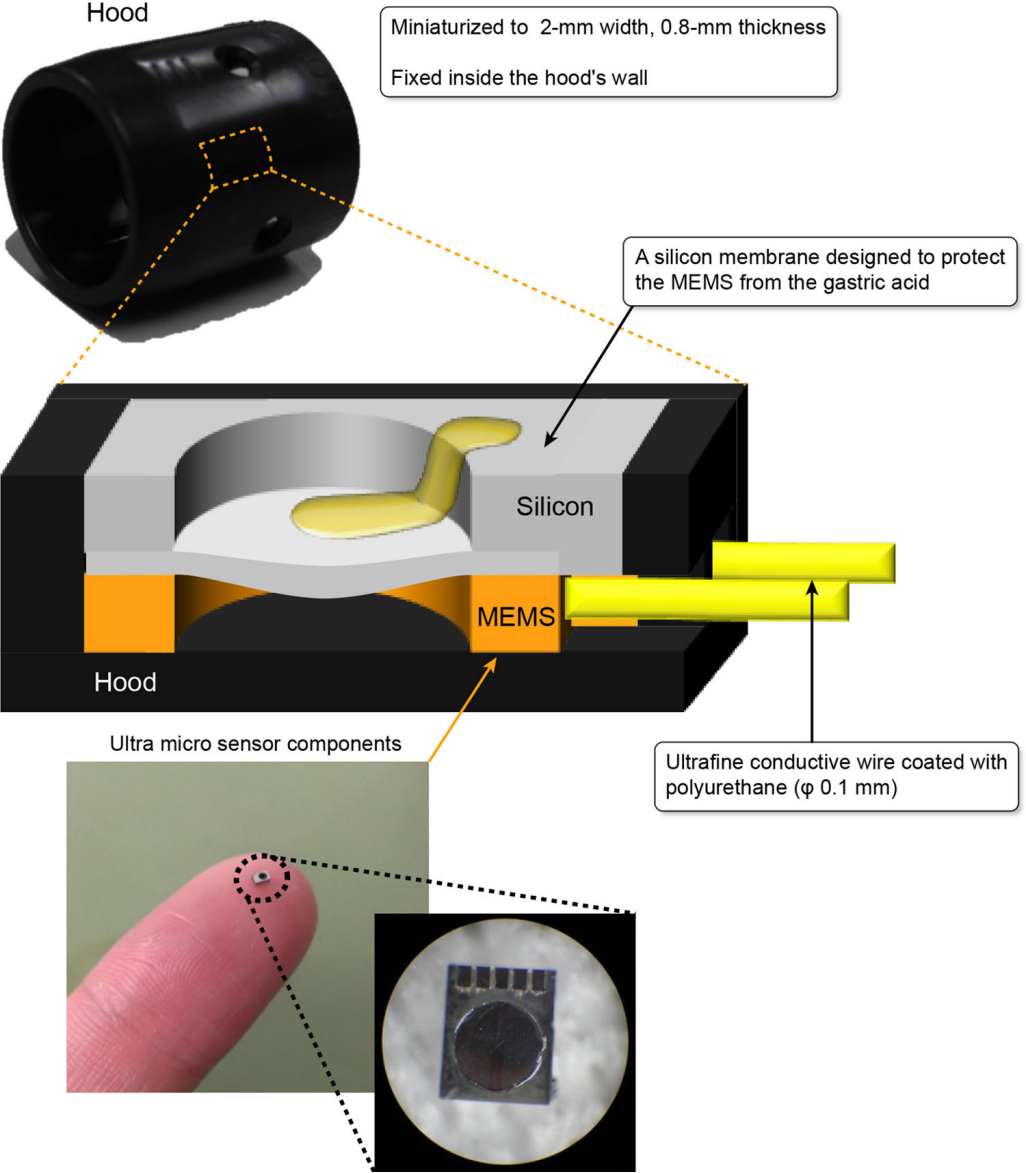
The embedding of an ultra-thin, small MEMS sensor inside the hood. The MEMS sensor was miniaturized to a 2-mm width and 0.8-mm thickness and was mounted onto a hood. The MEMS sensor was covered with a silicon membrane to protect it from gastric acid. The output originated from an ultrafine conductive wire coated with polyurethane

**Fig. 2:**
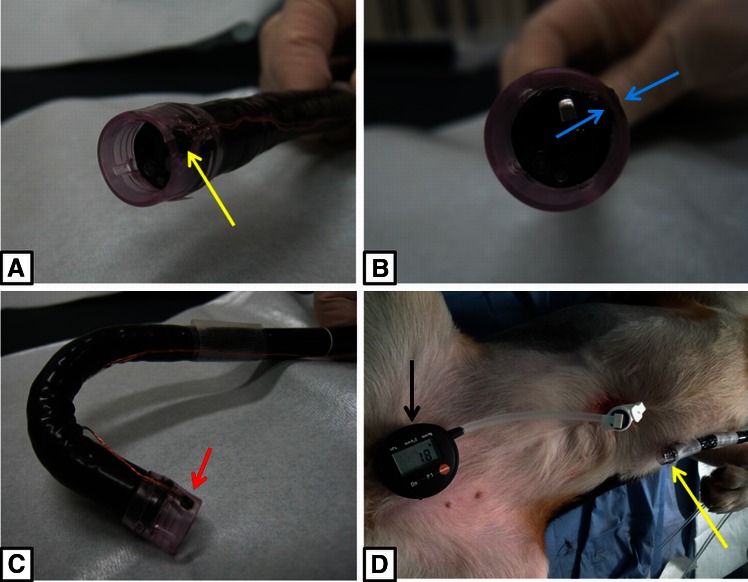
The external appearance of an endoscope equipped with an attached MEMS hood and the pressurization test. **A** Anterolateral view of the external appearance of the endoscope equipped with a MEMS hood. The *yellow arrow* indicates the miniaturized MEMS pressure sensor. **B** Anterior view of its external appearance. The MEMS is fixed inside the wall of the hood and does not interfere with the field of view of the endoscope. The *blue arrow* indicates the MEMS pressure sensor. **C** There was no restriction to the operability of the device, even when the endoscope was retroflexed. The *red arrow* indicates the MEMS pressure sensor. **D** We performed a percutaneous endoscopic gastrostomy (PEG) (Introducer method, Ideal PEG kit, Olympus Co., Tokyo, Japan) on a dog’s stomach to connect the stomach to the real-time pressure monitor outside the stomach (*black arrow*). The *yellow arrow* indicates the MEMS hood attached to the tip of endoscope

Figure 1 shows the MEMS sensor hood, which was miniaturized to a width of 2 mm and a thickness of 0.8 mm, and embedded into the wall of the hood. The MEMS pressure sensor did not interfere with the field of view of the endoscope, and the extremely fine polyurethane-coated conductive wire with a diameter of 0.1 mm did not cause any interference with the handling or operation of the endoscope. We coated the MEMS surface with a very thin membrane to protect it from the acid within the stomach. Figure 2A–C shows pictures of the sensor mounted on the hood and attached to the endoscope. We performed percutaneous endoscopic gastrostomy (PEG) (Introducer method, Ideal PEG kit, Olympus Co., Tokyo, Japan) on the dogs’ stomach to connect the stomach to the real-time pressure monitor outside the stomach (Fig. 2D).

First, the MEMS sensor hood was mounted on the tip of the endoscope, and air insufflation/deflation tests were performed for 20 consecutive cycles. In addition to the increase in the internal pressure caused by air insufflation from the endoscope, the correlation between the actual measured pressure and the signal strength of the MEMS hood was measured.

Next, we marked 2 points at intervals of 20 mm on the greater curvatures of the antrum, lower body, and middle body and on the lesser curvature of the lower body using an electric knife (dual knife, Olympus Co., Tokyo, Japan). Between two marks, we placed 20-mm-long measuring paper into the stomach to accurately measure the distances, even of the curved gastric wall. We gradually insufflated air until the maximum stable distance of the two markings was obtained. In each region, we performed five insufflation/deflation tests to obtain the relationship between the pressure and distance under every 2.5 mmHg intragastric pressure from 2.5 to 50–80 mmHg, and plotted it to measure the accurate distance in vivo in a dog’s stomach. The results of marked 2 points distances among the five insufflation/deflation tests under every 2.5 mmHg intragastric pressure until 50–80 mmHg were measured and analyzed.

### Experimental devices

Endoscopes: OLYMPUS GIF TYPE H260Z (Olympus Co., Tokyo, Japan). CO_2_ insufflation device: OLYMPUS UCR (Olympus Co., Tokyo, Japan). Percutaneous endoscopic gastrostomy (PEG) kit: Introducer method, Ideal PEG kit (Olympus Co., Tokyo, Japan). Marking knife: dual knife (KD-650L, Olympus Co., Tokyo, Japan). Generator device: ERBE VIO300D (Elektromedizin, Tübingen, Germany).

### Statistical analysis

The results of marked 2 points distances among the five insufflation/deflation tests under every 2.5 mmHg intragastric pressure from 2.5 to 50–80 mmHg were analyzed using Spearman’s correlation with the level of significance set at *P* < 0.05. Statistical analyses were performed using Graph Pad Prism version 5 for Windows (Graph Pad Software, San Diego, CA, USA).

## Results

**Fig. 3:**
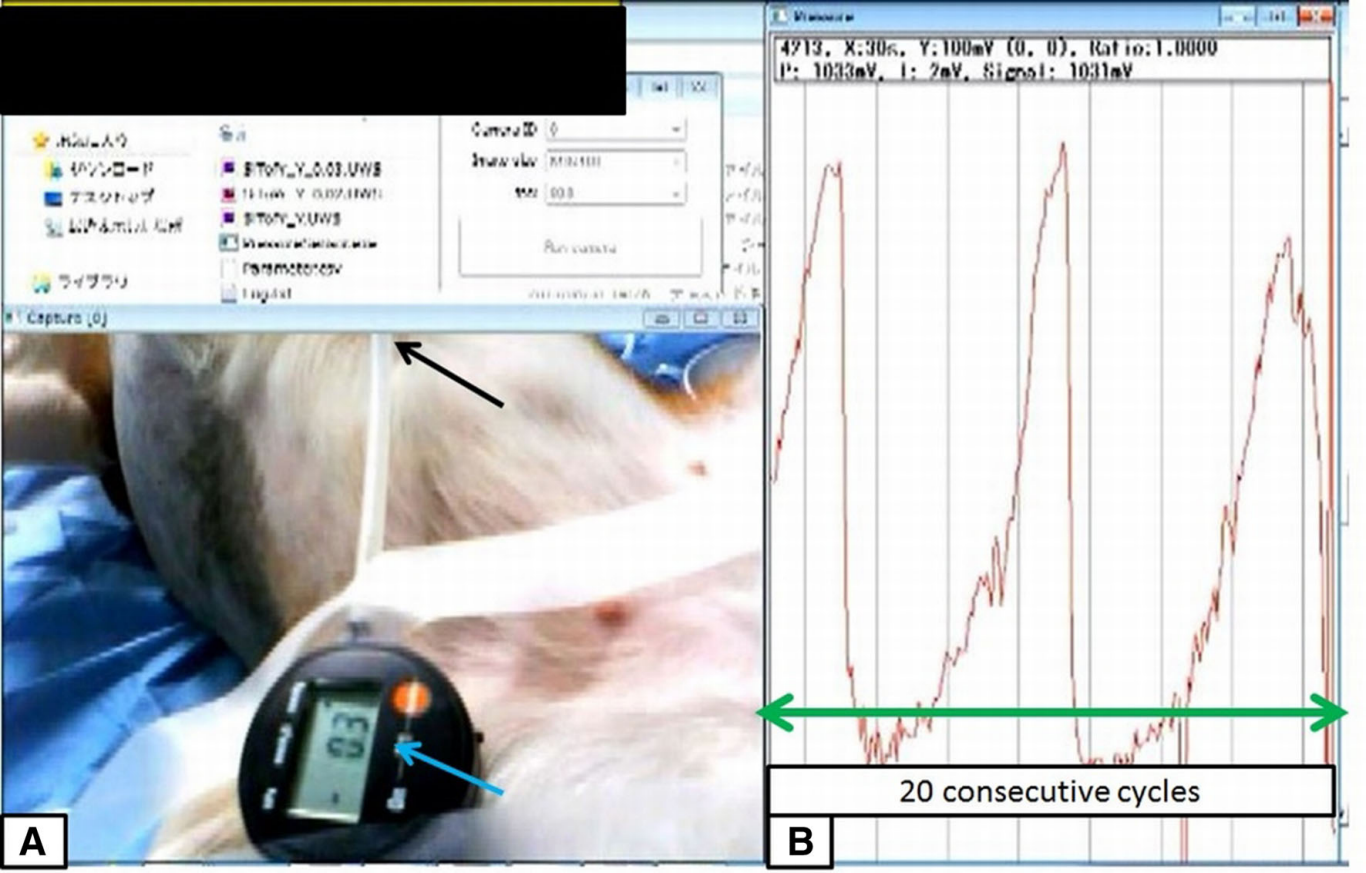
The pressurization/depressurization tests (air insufflation/deflation tests). **A** Pressurization/depressurization tests were performed 20 consecutive times. The *blue arrow* indicates the pressure measurement sensor, and the *black arrow* shows the PEG to the dog’s stomach. **B** In association with the elevation of the internal pressure due to the insufflation of air from the endoscope, we measured the correlation between the signal strength of the MEMS hood and the measured pressure for 20 consecutive cycles (*green arrow*)

**Fig. 4:**
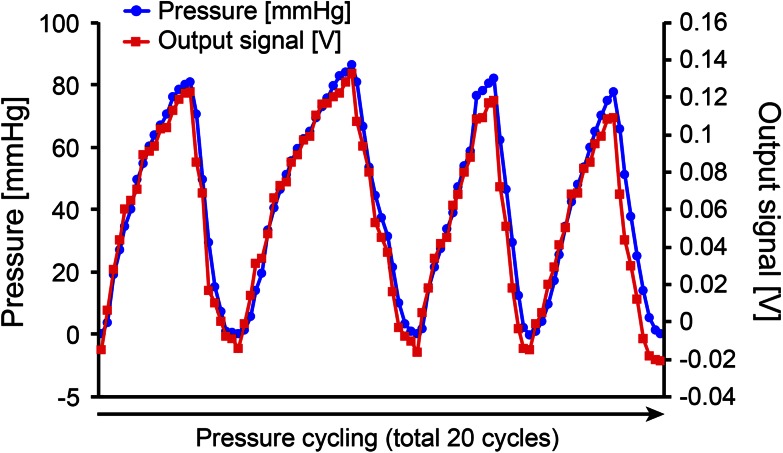
Time-course correlation between the MEMS output electrical signal and the measured internal pressure. A total of 20 cycles were measured accurately, with 4 cycles being shown. The output signal strength (V) of the MEMS hood and the pressure measurement sensor (mmHg) showed a good correlation. The signal strength from 20 cycles ranged from −0.01 to 0.138 V. The internal pressure measured by the pressure measurement sensor ranged from 0 to 83 mmHg, and a good correlation was found

Figure 3 and video 1 show the pressurization/depressurization tests (air insufflation/deflation test), which were performed 20 consecutive times. We found a time-dependent increase/reduction of the internal pressure that was similar to the findings from the pressure measurement sensor inserted from the PEG. When the pressure was increased to 80 mmHg, the MEMS hood detected the output signal strength in a time-dependent manner, and the output of the signal strength increased to a maximum of 0.14 V. The device was mounted on the tip of an endoscope, and the findings correlated well with the pressure measured by the pressure sensor from the PEG. The signal strength of the MEMS hood (V) and the values shown by the pressure measurement sensor (mmHg) showed a good correlation. The signal strength from 20 cycles ranged from −0.01 to 0.138 V. The internal pressure measured by the pressure measurement sensor ranged from 0 to 83 mmHg (Fig. 4).

**Fig. 5:**
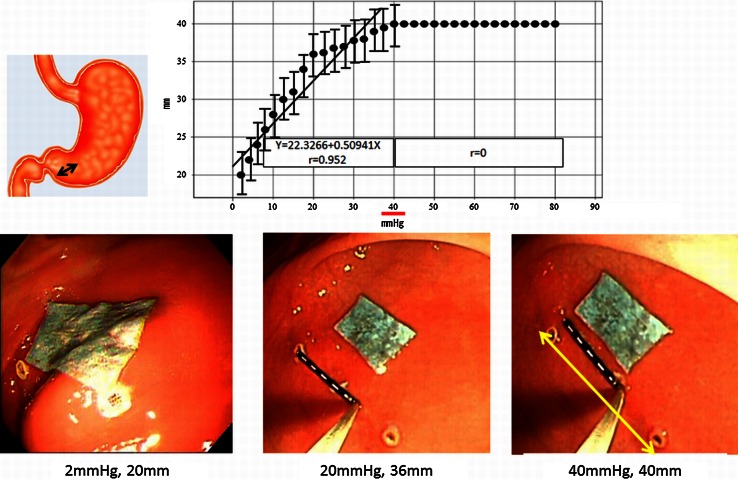
Air insufflation and expansion limit test of two markings in the greater curvature of the antrum. The correlation between pressure and the expansion limit of the distance between the two markings. In the greater curvature of the antrum, there were good correlations between intraluminal pressures in the range of 0–40 mmHg and the two marking distances (correlation coefficient 0.952). Once the intraluminal pressure reached a certain level (40 mmHg), the two marking distances ceased to expand further (40 mm)

Next, air was insufflated into the stomach until the expansion limit of the distance between the two markings was achieved. Figure 5 and video 2 show the correlation between air pressure and the expansion limit of the distance between the two markings. In the greater curvature of the antrum, there were good correlations between the intraluminal pressures in the range from 2.5 to 40 mmHg and the two marking distances (correlation coefficient 0.952) (*P* < 0.05). There were significant correlations until 40 mmHg. However, once the intraluminal pressure reached a certain level (40 mmHg), the two marking distances ceased to increase further.

**Fig. 6:**
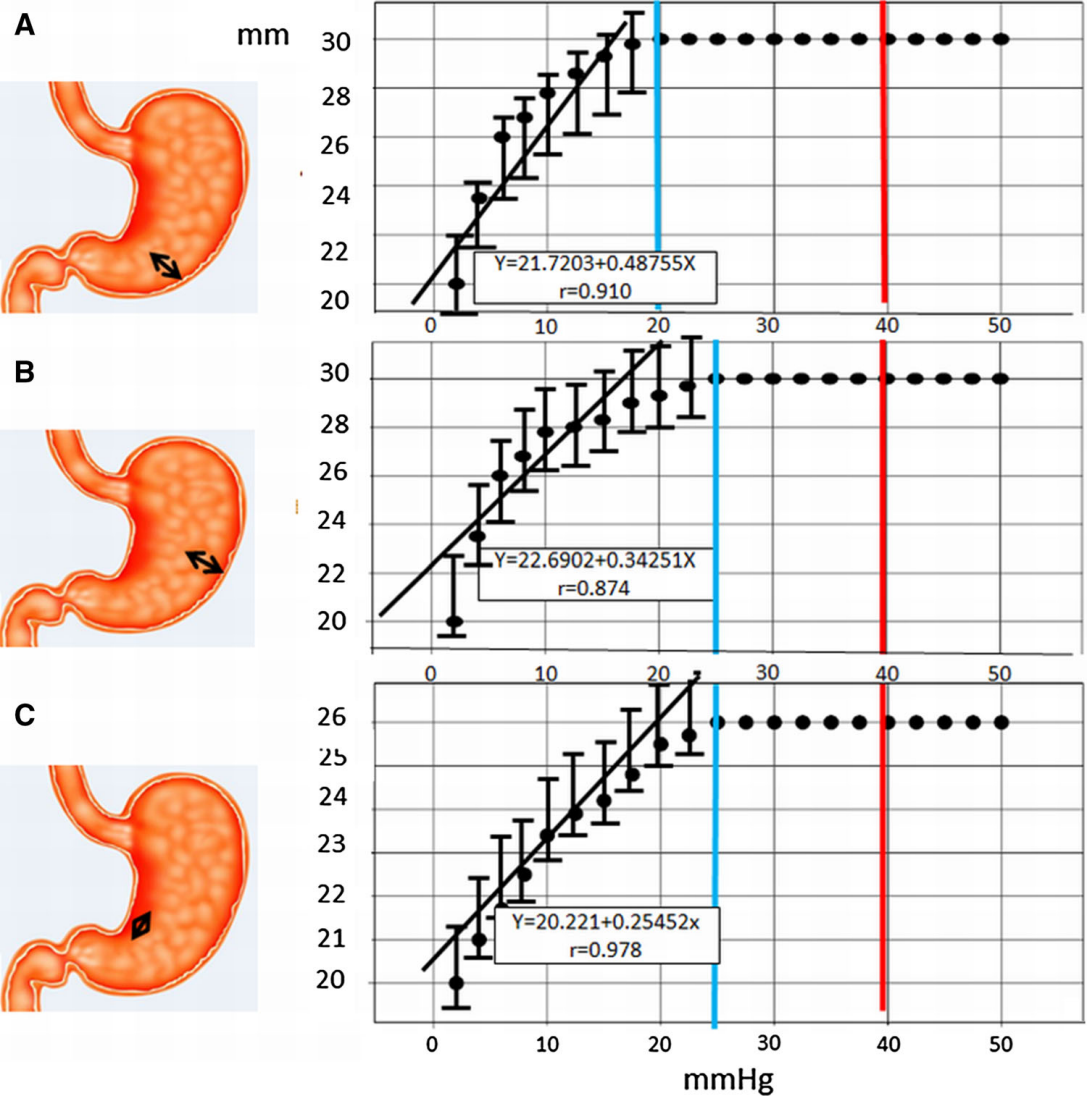
Air insufflation and expansion limit test of two markings in 3 points of the stomach. **A** The correlation between pressure and the expansion limit of the distance between two markings. In the greater curvature of the lower body, there were good correlations between intraluminal pressures in the range of 0–20 mmHg and the two marking distances (correlation coefficient 0.910). Once the intraluminal pressure reached a certain level (20 mmHg), the two marking distances ceased to expand further (30 mm). **B** In the greater curvature of the middle body, there were good correlations between intraluminal pressures in the range of 0–25 mmHg and the two marking distances (correlation coefficient 0.874). Once the intraluminal pressure reached a certain level (25 mmHg), the two marking distances ceased to expand further (30 mm). **C** In the lesser curvature of the lower body, there were good correlations between intraluminal pressures in the range of 0–25 mmHg and the two marking distances (correlation coefficient 0.978). Once the intraluminal pressure reached a certain level (25 mmHg), the two marking distances ceased to expand further (26 mm)

The same measurements were conducted in the greater curvatures of the lower body and middle body and on the lesser curvature of the lower body. In the greater curvature of the lower body, good correlations between pressure and distance were obtained from 2.5 to 20 mmHg (correlation coefficient 0.910, two marking maximum distance 30 mm) (*P* < 0.05). There were significant correlations (Fig. 6A). In the greater curvature of the middle body, good correlations between pressure and distance were obtained from 2.5 to 25 mmHg (correlation coefficient 0.874, two marking maximum distance 30 mm) (*P* < 0.05). There were significant correlations until 25 mmHg (Fig. 6B). In the lesser curvature of the lower body, good correlations between pressure and distance were obtained from 2.5 to 25 mmHg (correlation coefficient 0.978, two marking maximum distance 26 mm) (*P* < 0.05). There were significant correlations until 25 mmHg (Fig. 6C).

In these four sites, the maximal expanded distance between the two markings was 40 mm at 40 mmHg in the greater curvature of the antrum.

## Discussion

Most reports on air insufflation during the use of flexible endoscopes have mainly concentrated on the types of gases insufflated, specifically, carbon dioxide (CO_2_) [5–9].

In endoscopic therapy and in ESD, which requires a prolonged duration of treatment, CO_2_ use has been reported to be safe [10]. In particular, in colorectal ESD, the perforation rate has been reported to be high [11, 12]. However, reports have also shown that even if perforation occurs, CO_2_ insufflation enables the treatment to be conducted quickly, and the endoscopist can then use clips for closure [13–17].

The only report about the amount of gas insufflated during endoscopic tests or treatments involving the internal pressure inside the digestive tract detailed an ESD caused by air insufflation at an automatic constant pressure using an automatically controlled, steady pressure endoscopy (SPACE) [18]. Current flexible endoscopes have no pressure measurement functions, and the amount of air insufflated is estimated by endoscopists based on their subjective judgment and experience.

MEMS is a device that uses nanotechnology and in which an actuator and electronic circuit are integrated on a single silicon substrate. MEMS can be used to create an ultra-compact sensor capable of detecting pressure. By mounting this sensor in the hood, the pressure inside the digestive tract can easily be determined in real time, thereby enabling tests and treatments to be conducted objectively and safely. Moreover, the cost of MEMS is not overly high. The external device such as laparoscopic pneumoperitoneum apparatus is rather larger and more expensive than MEMS disposable hood, moreover, it is needed to upgrade the endoscope and endoscopic systems.

However, the most important point we should emphasize is that even in conventional EGD, the tumor size is measured subjectively using manual insufflation. As the pressure of the insufflated air changes the tumor size and shape, evaluations of tumor size and shape become more subjective, which may lead to misdiagnoses. In ESD, such an objective assessment has not yet been achieved, although lesion size is specifically mentioned as an important factor in the guidelines [19, 20]. The expanding indications of ESD are strictly defined by the Gastric Cancer Treatment guidelines of JGCA based on tumor size. However, depending on the amount of insufflated air, the tumor size measured can vary greatly. This process also lacks reproducibility and, as such, should not be used in quantitative indices or objective indices. If we define tumor size as an indication of ESD, more objective indices are absolutely essential as the size will affect the subsequent assessments of possible lymph node metastasis, recurrence, and patient prognosis [21]. Accordingly, the specimens must be accurately measured to ensure that the pathologists evaluate them correctly.

**Fig. 7:**
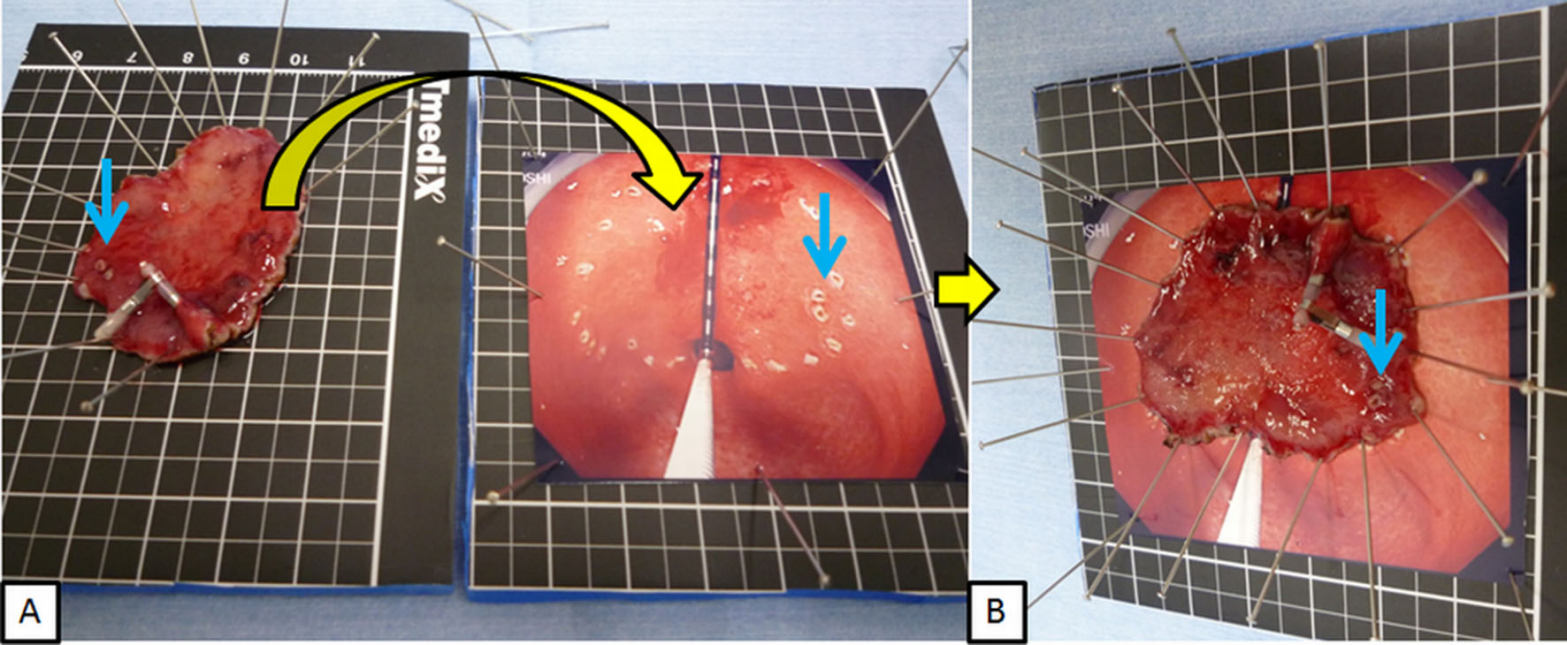
Method to evaluate correct tumor size under constant pressure in gastric ESD. **A** After marking the tumor under a certain constant pressure at which the tumor size does not expand, take the pictorial image from front of the tumor and print it out. Attach the image on the specimen board and put the resected specimen on the board (*yellow curved arrow*) according to two marking (*blue arrow*) and tumor margin of picture. **B** The resected specimen placed on the board accurately as it were in the dog’s stomach regarding size and shape of tumor

Before we discuss using tumor size as an indication of ESD, we should be able to accurately measure and evaluate the tumor size. To achieve this accuracy, we suggest the following methods. First, after marking the tumor with some markers under a certain constant pressure at which the tumor size does not expand, the pictorial image from the front of the tumor can be obtained and printed out. Next, the image should be attached to a specimen board (Fig. 7A), and the resected specimen placed on the board (Fig. 7B). As it might be assumed that the insufflation pressure under which the tumor size never expands differs among patients due to their physical status, we should perform an EGD to obtain the patient’s pressure information before the ESD.

The pressure inside the digestive tract can be determined in real time through the simple attachment of an MEMS hood, which will enable us to obtain an objective assessment in endoscopic diagnosis that will enable objective evaluations to be conducted. Additionally, in endoscopic therapy, the use of a MEMS hood may prevent the excessive insufflation of air, which will reduce the pain resulting from excessive insufflation for the patient during examinations or treatments. In our in vivo dogs’ experiments, MEMS hood could detect from the minimal pressure around 2 mmHg to the maximal pressure around 80 mmHg. Insufflations over 80 mmHg caused mucosal injury, mucosal oozing bleeding, and Mallory–Weiss syndrome of the stomach.

Ideally, the MEMS sensor should be mounted on the main body of the endoscope. However, in terms of medical economics, it would be difficult for all municipal and community hospitals to purchase endoscopes equipped with a MEMS. Alternatively, if the hood is placed at the tip, simply mounting the hood will facilitate the measurement of the pressure inside the gastrointestinal tract at a low cost. We are currently developing a wireless version of the MEMS hood in which the images are displayed in real time on the endoscope screen. We plan to conduct a prospective clinical trial of this device.

### Study limitations

This study was an in vivo animal study that included a small number of subjects. Therefore, prospective studies in clinical settings are still needed.

## Electronic supplementary material

Below is the link to the electronic supplementary material.

Supplementary material 1 (MPG 10364 kb)

Supplementary material 2 (MPG 10504 kb)
